# Green Tea Consumption and Risk of Breast Cancer and Recurrence—A Systematic Review and Meta-Analysis of Observational Studies

**DOI:** 10.3390/nu10121886

**Published:** 2018-12-03

**Authors:** Vincenza Gianfredi, Daniele Nucci, Angela Abalsamo, Mattia Acito, Milena Villarini, Massimo Moretti, Stefano Realdon

**Affiliations:** 1 Post-Graduate School of Hygiene and Preventive Medicine, Department of Experimental Medicine, University of Perugia, P.le L. Severi 1, 06132 Perugia, Italy; 2 Digestive Endoscopy Unit, Veneto Institute of Oncology IOV-IRCCS, Via Gattamelata 64, 35128 Padua, Italy; stefano.realdon@iov.veneto.it; 3 Department of Pharmaceutical Science, University of Perugia, Via del Giochetto 2, 06123 Perugia, Italy; angela.abalsamo@yahoo.it (A.A); mattia.acito@studenti.unipg.it (M.A.); milena.villarini@unipg.it (M.V.); massimo.moretti@unipg.it (M.M.)

**Keywords:** breast cancer risk, green tea, epigallocatechin, women, cancer recurrence, *Camellia sinensis*, meta-analysis

## Abstract

Breast cancer (BC) is the most common cancer in women and several factors are involved in its onset. Green tea (GT) has been shown to have potential beneficial effects on different types of cancer. The aim of this review was to evaluate the association between GT regular consumption and risk of BC in women. The risk of BC recurrence and risk of BC in relation to menopausal status were also evaluated. A literature search of PubMed, Scopus, and Web of Science was conducted. Preferred Reporting Items for Systematic Reviews and Meta-Analyses (PRISMA) guidelines were followed to perform the systematic review and meta-analysis. Full texts were downloaded for 40 studies; however, only 13 records were included in the meta-analysis. Eight were cohort studies and five were case-control studies. The pooled sample consisted of 163,810 people. An inverse statistically significant relationship between GT and BC risk, with an Odds Ratio (OR) = 0.85 ((95% CI = 0.80–0.92), *p* = 0.000)), was found. Egger’s linear regression test did not show a potential publication bias (intercept 0.33, *t* = 0.40, *p* = 0.695), which was also confirmed by the symmetry of the funnel plot. Moreover, no high statistical heterogeneity (Chi^2^ = 31.55, *df* = 13, *I*^2^ = 58.79%, *p* = 0.003) was found. The results of this meta-analysis showed a potential protective effect of GT consumption on BC, especially for BC recurrence.

## 1. Introduction

Tea is one of the most consumed beverages worldwide. It is obtained from leaves and buds of *Camellia sinensis* (*C. sinensis*). The fruit of *C. sinensis* is three-celled and green in color, and the seed is a brown, semi-globose nut [1,2]. Young and light green leaves, as well as new shoots, are generally harvested to produce tea. After harvesting, various techniques of processing tea leaves lead to three main types of tea, characterized by different polyphenols content, from both a qualitative and quantitative point of view, and by different flavors.

Green tea is produced by exposing fresh leaves to heat or hot steam immediately after plucking, and this results in minimal polyphenol oxidation. If fresh leaves are allowed to stand for an hour (or less) before heat treating, the final product is called oolong tea, characterized by an intermediate stage of oxidation between green and black tea. The latter is obtained by a series of processes, including withering, pre-conditioning, “cut-tear-curl”, fermentation, where the leaves are exposed to the air, and final drying [3,4].

Initially, tea was traditionally used in a popular Chinese medicine, and only later did it spread as a beverage. Its history is intricate, and its origin is still uncertain. Some legends claim it was discovered in China by Emperor Shennong, around 5000 years ago; others affirm it originated in India and was later brought to China, Japan, and Korea thanks to the spread of Buddhism [5]. One of the earliest written references dates back to 221 B.C., when the Chinese Emperor Qin Shi Huang signed a document to introduce a tax on tea, while the first handbook was written by Lu-Yu in the eighth century [6]. Tea was first introduced in the Western World by the Turks (around 600 AD), but only in the seventeenth century did it began to spread all over Europe, thanks to Dutch and English imports from Asia (India, Sri Lanka, China, Japan, Indonesia) [5,6,7]. Nowadays, tea is produced in several countries: the main producer is China (1.9 million metric tons in 2013), followed by India, Kenya, and Sri Lanka [8].

The potential beneficial effects of tea are due to the large amount of bioactive compounds (approximately 4000), one-third of which belong to the polyphenol group [7]. Catechins are the most important polyphenol class in tea. Tea also contains flavonols, phenolic acids, and methylxanthines [9,10]. Among teas, different production techniques translate into different phytochemical characteristics. Green tea is the most studied for its potential benefits, since it retains a major amount of catechins in comparison to other teas. Indeed, catechins constitute 30–42% of the total dry weight of green tea [10]. The most abundant catechins are (–)-epigallocatechin-3-gallate (EGCG), (–)-epigallocatechin (EGC), (–)-epicatechin-3-gallate (ECG), and (–)-epicatechin (EC), which represent approximately 59%, 19%, 13%, and 6.4% of all catechins, respectively [11]. Several studies have demonstrated preventive and likely chemotherapeutic activities of green tea polyphenols and EGCG against breast, skin, colon, lung, prostate, liver, and stomach cancer [12,13,14,15,16,17,18]. However, the molecular mechanisms are still unknown. Among the most plausible mechanisms involved, antioxidant activity, inhibition of clonal expansion of cancer stem cells by the maintenance of a quiescent state, downregulation of oncogenes and upregulation of tumor-suppressor genes through epigenetic phenomena, regulation of telomerase activity, and modulation of membrane lipid raft have been investigated [19,20,21,22]. Recently, green tea EGCG has been shown to inhibit angiogenesis through different pathways and mechanisms such as modulation of cell proliferation, suppression of angiogenic factors, and induction of apoptosis [20,21]. Moreover, miRNAs might be involved in the process via the inhibition of binding of vascular endothelial growth factor (VEGF) to its receptor [23]. In particular, previous studies highlighted the role played by EGCG on epigenetic modification of genes involved in early carcinogenesis and/or breast cancer progression. Among females, breast cancer (BC) is the most frequent cancer, with the highest cancer mortality rate among women worldwide [24]. According to GLOBOCAN, in 2018, BC will represent almost 1 in 4 of cancer cases among women, with 2.1 million new diagnoses [24]. Moreover, BC incidence is higher in Europe and North America compared to Asia and Africa. Although well-known hereditary and genetic factors are involved in BC aetiology, the increased risk among low-risk populations migrating into high-risk areas revealed the potential role played by other factors such as oral contraceptive drugs, parity, menstruation, physical activity, breast feeding, and nutrition [25]. Previous studies provided limited/non-conclusive evidence on the correlation between green tea consumption and the risk of BC. The aim of this systematic review and meta-analysis was to evaluate the association between green tea regular consumption and risk of BC in women. Furthermore, the risk of BC recurrence and risk of BC in relation to menopausal status was assessed.

## 2. Material and Methods

Preferred Reporting Items for Systematic Reviews and Meta-Analyses (PRISMA) guidelines were followed to perform the systematic review and meta-analysis [26]. A literature search was conducted on 18 September 2017 and updated on 19 November 2018 in PubMed, Scopus, and Web of Science. The databases were searched using appropriate key words, pre-determined based on the type of the database consulted. In PubMed/Medline, a combination of Medical Subject Headings (MeSH) terms, title/abstract, and all field were used. In both databases, the search terms were selected based on three aspects: green tea consumption, breast cancer risk, and type of study. The keywords were then combined using Boolean operator AND/OR. Considering the listed aspects, the search strategy for PubMed/Medline was:

-green tea consumption: “green tea”[Title/Abstract] OR “Camellia sinensis”[MeSH] OR “Tea”[MeSH] OR “green tea”[Title/Abstract] OR “Polyphenols”[MeSH] OR “epigallocatechin gallate”[Supplementary Concept];

-breast cancer risk: ((“breast neoplasms”[MeSH Terms] OR breast cancer[Text Word] OR breast cancer[Title/Abstract] OR ((“breast”[MeSH Terms] OR “breast”[All Fields]) AND tumor[Title/Abstract];

-study design: “Case-Control Studies”[MeSH] OR “Cohort Studies”[MeSH] OR “Retrospective Studies”[MeSH] OR “Prospective Studies”[MeSH].

For searches on Scopus, the following search terms were used:

-green tea consumption: “green tea” OR “Camellia sinensis” OR Tea OR epigallocatechin gallate OR Polyphenols

-breast cancer risk: breast neoplasm OR breast cancer OR breast tumor

-study design: “Case-Control Studies” OR “Cohort Studies” OR “Retrospective Studies” OR “Prospective Studies”.

### 2.1. Inclusion Criteria

Articles included in the current meta-analysis met the following criteria: studies performed on humans, research focused on green tea consumption, full text available, epidemiologic studies, only articles in English. Inversely, exclusion criteria were: studies with different outcomes, data not reported as risk (for instance Odds Ratio - OR; Relative Risk - RR; Hazard Ratio - HR) or articles without original data, and animal model trials. As the aim of this review was not to evaluate the effectiveness of an intervention in a population, randomized controlled trials (RCTs) were not included [27]. Furthermore, green tea drinking is a common human practice so it is difficult to evaluate by experimental study design. Indeed, all the RCTs retrieved during the reference screening evaluated the effect of green tea extract supplementation instead of green tea consumption. Details of inclusion/exclusion criteria are presented in Table 1, according to PICOS (Population, Intervention, Comparison, Outcome, Study type) format expanded with time filter and language [28].

To validate the inclusion of the retrieved studies, two reviewers (V.G. and A.A.), independently and blinded, screened titles and abstracts. Possible disagreements were resolved through discussion or third reviewer consultation (D.N.). The reviewers read the full texts of the selected articles and double-checked the list of references of the selected studies in order to detect any other potentially relevant papers.

### 2.2. Data Extraction

Data were collected independently by two authors (V.G. and A.A.) and reported in a pre-determined spreadsheet. Extracted data contained both qualitative and quantitative information. In particular, qualitative data included the name of the first author, year of publication, type of study, and country where the study was conducted. Quantitative study design characteristics, namely data on enrolment, exposure, tools used to estimate food intake (for instance, Food Frequency Questionnaire (FFQ), validated or not, with the FFQ either self-administered or by interview), and information on the outcomes, were also collected. Furthermore, participant characteristics such as sample size, age range or mean age, and health status were also collected.

### 2.3. Quality Evaluation

In order to perform a sensitivity analysis, the quality of the included studies was also evaluated by two authors (A.A. and M.A.) using the scoring system created on the basis of Meta-analysis of Observational Studies in Epidemiology (MOOSE) [27], Quality Assessment Tool for Systematic reviews of Observational studies (QATSO) [29], and STrengthening the Reporting of OBservational studies in Epidemiology (STROBE) [30] and modified by Buitrago-Lopez et al., who applied the scoring tool on food intake and risk of chronic diseases [31]. The scoring system assigns zero or one point based on the evaluation of the six variables considered. The highest score available is six and the variables analyzed are: (*i*) justification given for the cohort (at least 80% of the initial included participants); (*ii*) appropriate inclusion and exclusion criteria; (*iii*) histological diagnosis of breast cancer; (*iv*) validated tool to assess green tea consumption; (*v*) adjustments were made for age, sex, body mass index, smoking status, and parity; and (*vi*) any other adjustments (such as for dietary factors, age at menarche, menopausal status, alcohol consumption).

### 2.4. Statistical Analysis

The software ProMeta 3 (Internovi, Cesena, Italy) was used to run the meta-analysis. The effect size (ES) was estimated by odds ratio (OR) reported with its 95% confidence interval (CI). For this study, *p* < 0.05 was considered statistically significant. The statistical heterogeneity among studies was assessed by the Chi^2^ test and *I*^2^ statistic (considering a value *I*^2^ > 60% as being statistically heterogeneous) [32]. To calculate the pooled effect, a fixed effect model was applied according to the found heterogeneity (Egger’s linear regression test). Lastly, a funnel plot was visually evaluated to assess possible publication bias. In addition, the plot of publication year bias was also evaluated.

The meta-analysis was conducted considering the risk of breast cancer among participants exposed to the highest versus the lowest consumption of green tea, as reporting of the frequency, dose, and unit (mL, g, cups, etc.) of tea consumption varied among the original studies.

### 2.5. Sensitivity Analysis

In order to corroborate the strength of the results, an additional subgroup analysis was performed, taking into account quality score, study design, risk of BC, and recurrence.

## 3. Results

### 3.1. Literature Search

**Figure 1:**
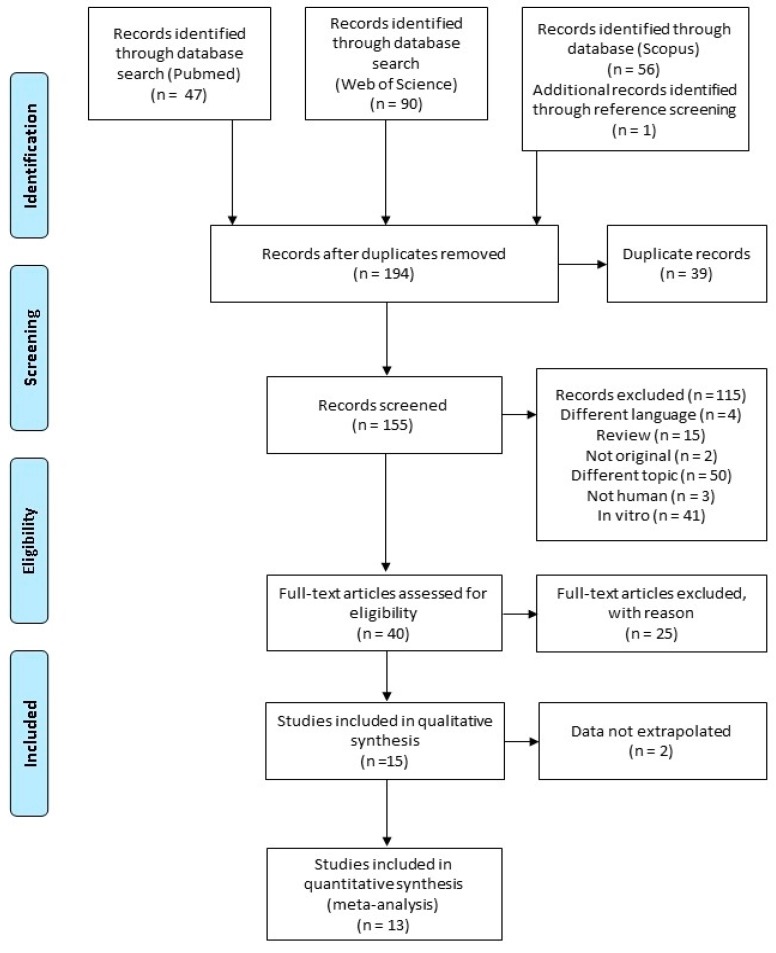
Flow diagram of studies selection process.

From PubMed/Medline, 47 articles were retrieved, whilst 56 articles were found in Scopus and 90 in Web of Science. After bibliography list screening, one more article was added, giving a total of 194 potentially relevant articles. However, 39 studies were duplicates and immediately eliminated. After further screening, based on evaluation of title and abstract, another 115 records were excluded, as reported in Figure 1. Full texts were downloaded for the remaining 40 studies. A further 25 studies were excluded for various reasons, mainly because no differentiation between green tea and other teas was taken into account (see Table S1 in the Supplementary Materials) [33,34,35,36,37,38,39,40,41,42,43,44,45,46,47,48,49,50,51,52,53,54,55,56,57]. The remaining 15 studies were included for quality synthesis. Nevertheless, two other studies were eliminated because data were not extrapolated [58,59]. At the end of the screening flow, 13 records were included in the meta-analysis. However, because Suzuki et al. reported the risk of BC in two different cohorts, this paper was considered as two independent studies [60].

### 3.2. Characteristics of Included Studies

**Figure 2:**
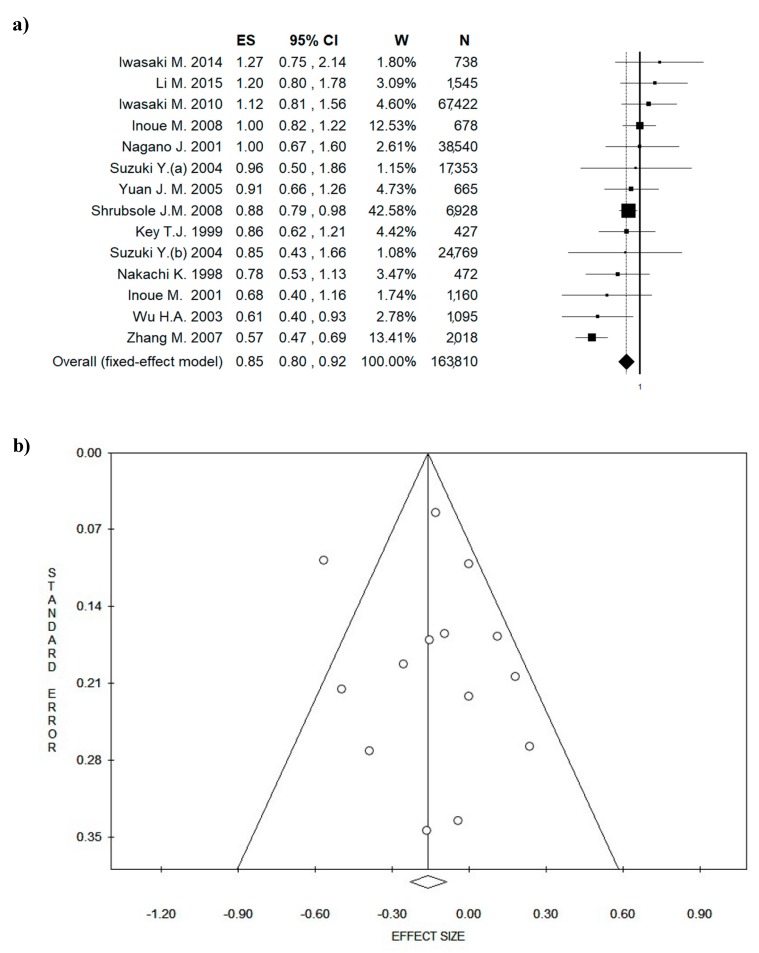

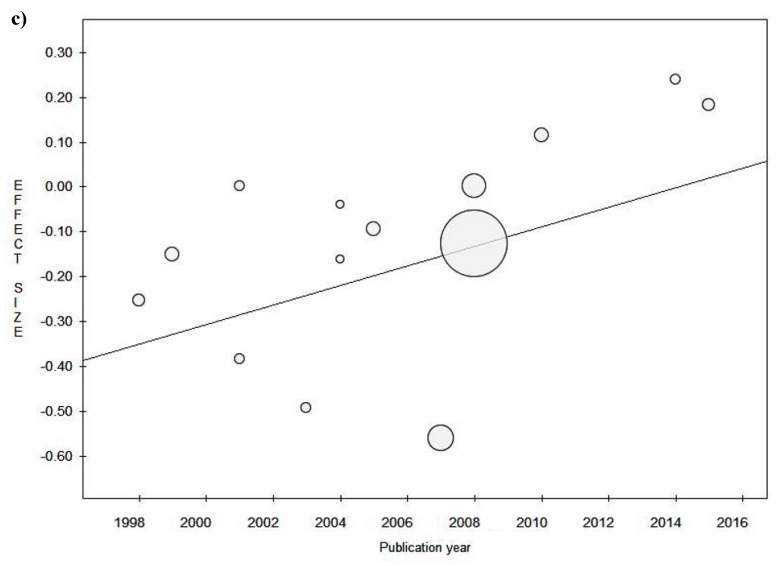
Forest plot (**a**) of the meta-analysis comparing green tea consumption in breast cancer (BC). Funnel plot (**b**). Publication year plot (**c**). Abbreviations: ES = effect size; CI = confidence interval; W = weight; Sig = significance; N = number.

All the studies included in the analysis are tabulated in Table 2. Looking at the country where the studies were performed, seven studies were conducted in Japan [60,61,62,63,64,65,66], five in China [67,68,69,70,71], and only one in the United States of America (USA) [72]. The age of the participants ranged between 20–87 years. Moreover, almost all the studies were relatively recent; only two studies were conducted before 2000 [64,66]. Around half of the studies were cohort studies [60,61,62,64,65,66,67,70] and five were case-control studies [63,68,69,71,72]. Moreover, seven studies were conducted on women with diagnosis of BC analyzing the risk of BC recurrence [61,63,66,68,69,71,72], whilst six studies recruited healthy participants and estimated the risk of BC [60,62,64,65,67,70]. The pooled sample consisted of 163,810 people and the ES was 0.85 ((95% CI = 0.80–0.92), *p* = 0.000)) (Figure 2a). The results of this meta-analysis show an important effect in the highest vs. the lowest category of green tea consumption; moreover, Egger’s linear regression test does not show a potential publication bias (intercept 0.33, *t* = 0.40, *p* = 0.695), also confirmed by the symmetry of the funnel plot (Figure 2b). No high statistical heterogeneity (Chi^2^ = 31.55, *df* = 13, *I*^2^ = 58.79%, *p* = 0.003) was found. Figure 2c shows the potential bias due to year of publication.

### 3.3. Sensitivity Analysis

#### Study Design

**Figure 3:**
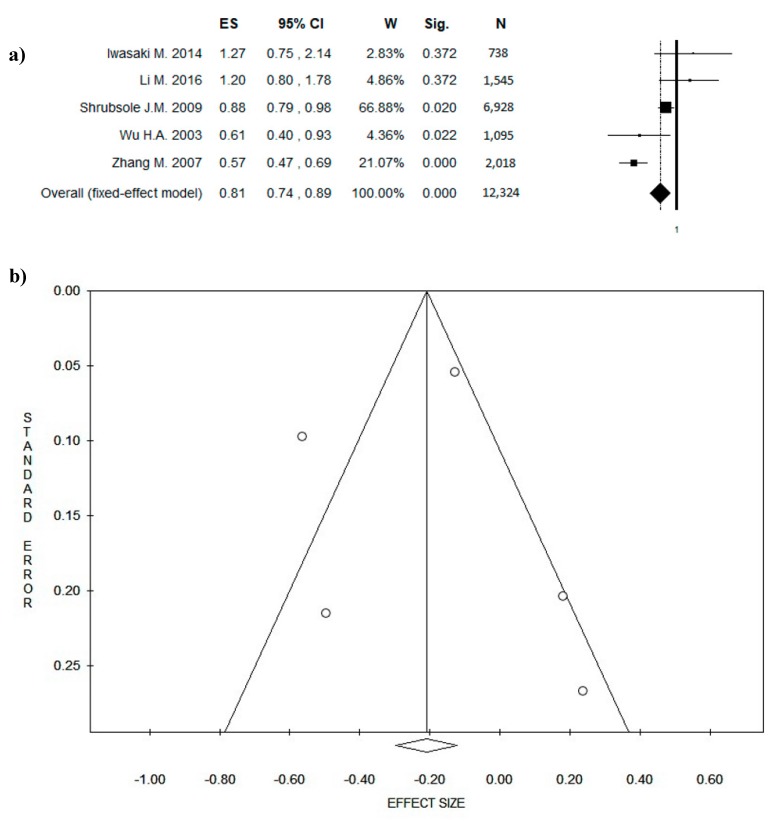
Forest plot (**a**) of the meta-analysis comparing green tea consumption in breast cancer (BC) prevention (case-control studies). Funnel plot (**b**). Abbreviations: ES = effect size; CI = confidence interval; W = weight; Sig = significance; N = number.

Any potential change in the results was analyzed, firstly performing the analysis according to the original studies design. When only cohort studies were included in the meta-analysis, the selection ranged from 472 to 67,422 participants followed up for 6 years on average. All the studies recruited healthy participants except for two papers that selected women with BC diagnosis. Considering all the cohort studies, the pooled sample size consisted of 151,486 participants with ES = 0.94 ((95% CI = 0.83–1.05), *p* = 0.273)) and with low potential publication bias (intercept −0.90, *t* = −1.38, *p* = 0.209, statistical heterogeneity Chi^2^ = 4.3, *df* = 8, *p* = 0.822, *I*^2^ = 0.00, *T*^2^ = 0.00). However, when the two studies that enrolled women with BC were excluded, the results did not change significantly ES = 0.97 ((95% CI = 0.86–1.11), *p* = 0.273)) (intercept −0.38, *t* = −0.69, *p* = 0.521, statistical heterogeneity Chi^2^ = 1.64, *df* = 6, *p* = 0.950, *I*^2^ = 0.00, *T*^2^ = 0.00). Inversely, after selective inclusion of only case-control studies, the pooled sample size consisted of 12,324 participants with ES = 0.81([(95% CI = 0.74–0.89), *p* = 0.000)) and with no potential publication bias (intercept 0.12, *t* = 0.05, *p* = 0.962), even though a statistical heterogeneity was found (Chi^2^ = 23.42, *df* = 4, *p* = 0.000, *I*^2^ = 82.92, *T*^2^ = 0.08); probably due to a low number of included studies. Data are shown in Figure 3a (forest plot) and 3b (funnel plot).

### 3.4. Outcome: BC Diagnosis and BC Recurrences 

**Figure 4:**
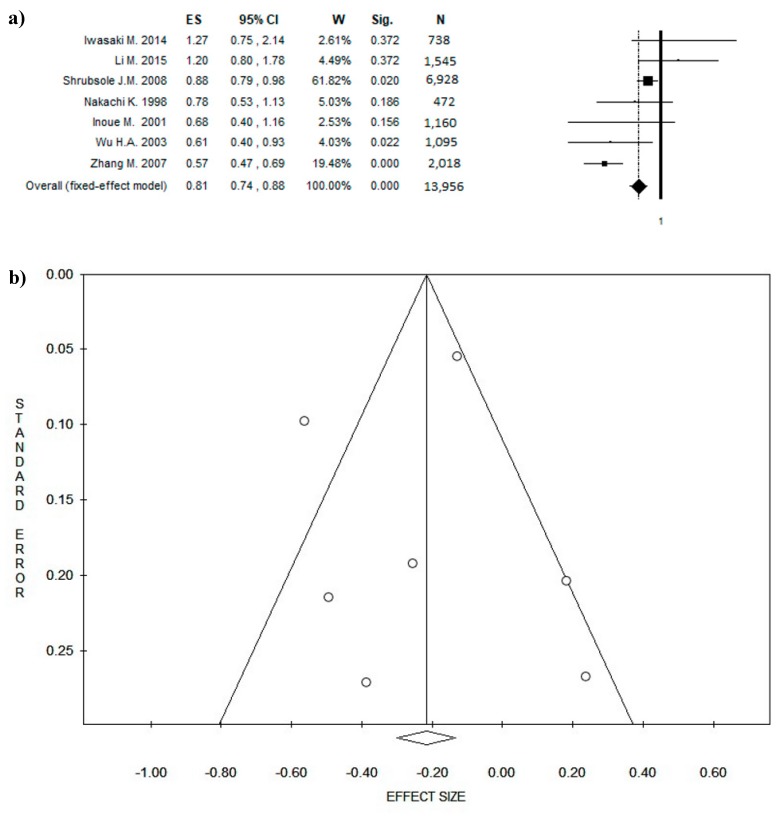
Forest plot (**a**) of the meta-analysis comparing green tea consumption in breast cancer (BC) recurrence. Funnel plot (**b**). Abbreviations: ES = effect size; CI = confidence interval; W = weight; Sig = significance; N = number.

Seven studies focused on the risk of BC recurrence [61,63,66,68,69,71,72]: one was conducted in USA, three in China, and the remaining in Japan. In three of these studies, the ESs indicated a potential protective effect of BC recurrence, while in the other four papers, the reduction was not statistically significant. The pooled ES was 0.81 ((95% CI = 0.74–0.88), *p*-value = 0.000) based on a total of 13,956 participants, with participant numbers in individual studies ranging from 472 to 6928 participants (Figure 4a). A slight statistical heterogeneity was found (Chi^2^ = 23.87, *df* = 6, *I*^2^ = 74.56%, *p* = 0.001). The funnel plot (Figure 4b) shows no potential publication bias, which was confirmed by Egger’s linear regression test (Intercept −0.16, *t* = −0.11, *p* = 0.915).

A supplementary analysis of the studies focusing on the risk of new BC diagnosis was conducted. In this case, none of the included studies reported a statistically significant ES, which was also confirmed by the pooled ES of the current meta-analysis. Indeed, the combined ES was 0.97 ((95% CI = 0.86–1.11), *p*-value = 0.684). However, no statistical heterogeneity (Chi^2^ = 1.64, *df* = 6, *I*^2^ = 0.00%, *p* = 0.950) was found (data not shown). Moreover, only three studies reported the stage of BC. In particular, Inoue et al. 2001 and Iwasaki et al. 2014 [63] only included women in the first and second stage of BC. In this analysis, ES was 1.0 ((95% CI = 0.90–1.10), *p* = 0.926) based on 1898, participants with no statistical heterogeneity (Chi^2^ = 2.69, *p* = 0.101, *I*^2^ = 62.76%).

### 3.5. Quality Score

**Figure 5:**
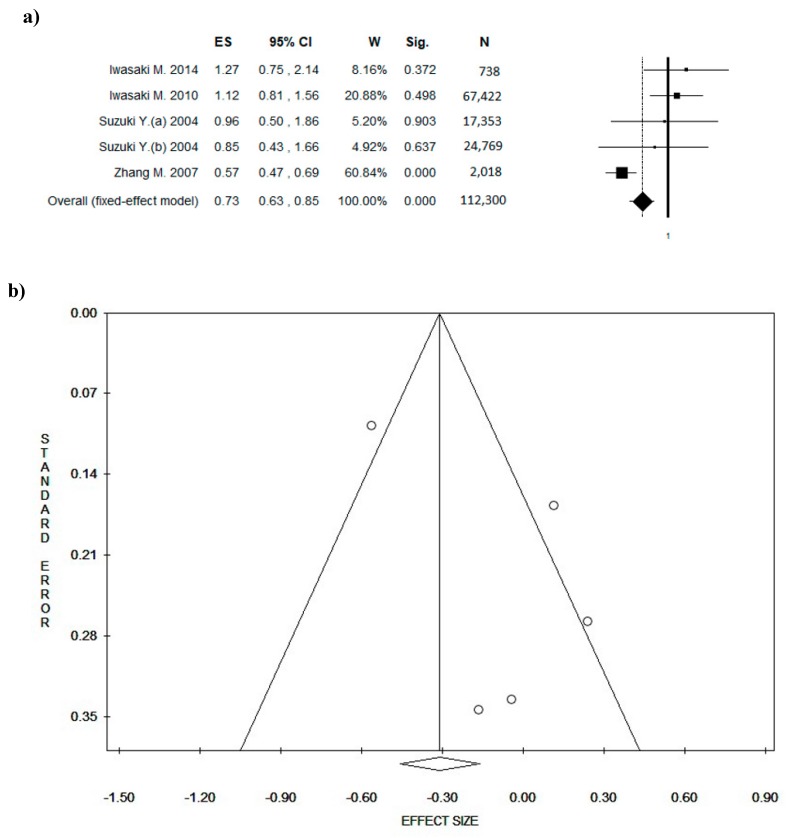
Forest plot (**a**) of the meta-analysis comparing green tea consumption in breast cancer (BC) (studies with quality score ≥ 5). Funnel plot (**b**). Abbreviations: ES = effect size; CI = confidence interval; W = weight; Sig = significance; N= number.

For a more comprehensive evaluation, a meta-analysis of studies with QS ≥ 5 was performed. In this case, ES was 0.73 ((95% CI = 0.63–0.85), *p*-value = 0.000) based on a total of 112,300 participants, with the number of participants for individual studies ranging from 738 to 67,422 participants (Figure 5a). A slight statistical heterogeneity was found (Chi^2^ = 18.08, *df* = 4, *I*^2^ = 77.87%, *p* = 0.001). The funnel plot (Figure 5b) shows potential publication bias, which was confirmed by Egger’s linear regression test (intercept 2.90, *t* = 1.79, *p* = 0.172).

### 3.6. Amount of Green Tea Intake

In order to find a potential beneficial amount of green tea, another meta-analysis of the studies reporting 5 cups of tea/day was performed. In this case, ES was 0.97 ((95% CI = 0.81–1.18), *p*-value = 0.783) based on a total of 148,511 participants, with the number of participants for individual studies ranging from 427 to 67,422 participants. No statistical heterogeneity was found (Chi^2^ = 1.46, *df* = 5, *I*^2^ = 0.00%, *p* = 0.917), and the lack of potential publication bias was confirmed by Egger’s linear regression test (Intercept = −-0.23, *t* = −0.62, *p* = 0.571).

### 3.7. Menopausal Status

A meta-analysis looking at menopausal status was also performed (Table 3). 

**Figure 6:**
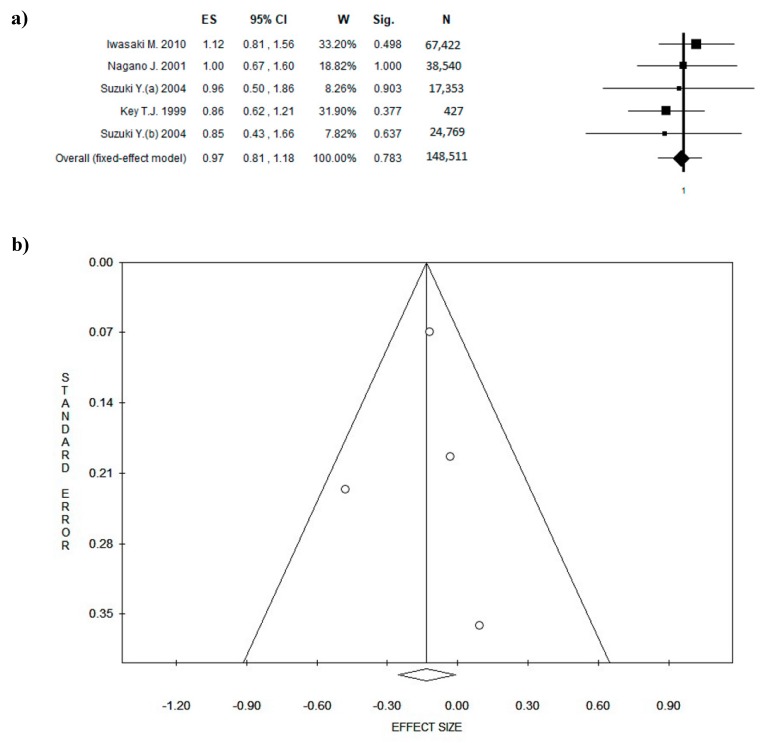
Forest plot (**a**) of the meta-analysis comparing green tea consumption in breast cancer (BC) (women in pre-menopausal status). Funnel plot (**b**). Abbreviations: ES = effect size; CI= confidence interval; W = weight; Sig = significance; N = number.

The risk of BC in women before and after menopause was compared. In the analysis, green tea intake showed a statistically significant protective role in pre-menopausal women. In this case, ES was 0.88 ((95% CI = 0.78–0.99), *p*-value = 0.035) based on a total of 1729 participants, with participant numbers for individual studies ranging from 79 to 1302 participants (Figure 6a). No statistical heterogeneity was found (Chi^2^ = 3.04, *df* = 3, *I*^2 ^ = 1.45%, *p* = 0.385), with the lack of potential publication bias confirmed by Egger’s linear regression test (intercept = −0.18, *t* = −0.16, *p* = 0.887) (Figure 6b). Inversely, in post-menopausal women, no effect was found. ES was 1.10 ((95% CI = 0.85–1.43), *p*-value = 0.474) based on a total of 1486 participants, with participant numbers for individual studies ranging from 70 to 799 participants. No statistical heterogeneity was found (Chi^2^ = 7.166, *df* = 3, *I*^2^ = 58.09%, *p* = 0.067), with potential publication bias confirmed by Egger’s linear regression test (intercept = 2.47, *t* = 2.04, *p* = 0.179).

## 4. Discussion

The potential beneficial effect of green tea could be related to its high amount of natural phytochemicals, which are preserved because of the absence of fermentation during the production process. Among green tea phytochemicals, polyphenols are the most represented. Several experimental studies have shown that long-term exposure to polyphenols may reduce chronic inflammation and oxidative stress and inhibit the growth, reproduction, and diffusion of cancer cells [73,74]. These experimental studies suggest a potentially protective effect of green tea consumption.

This meta-analysis showed the potential effect of green tea consumption in the reduction of breast cancer risk (15%). Furthermore, because the CI shows the interval within which a true mean is likely to be found, the narrow CIs observed in this systematic review may suggest the precision of ES results. In a sensitivity analysis of case-control studies and those with QS ≥ 5, a higher protective effect of green tea was found. In particular, a 19% reduction for case-control studies and a 27% reduction for studies with QS ≥ 5 were found. 

The majority of cohort studies included in this meta-analysis found a significant reduction of BC recurrence, although data was not confirmed in the case-control studies. The presented results highlight the important role of green tea in tertiary prevention rather than in primary prevention. Actually, green tea appears to reduce BC incidence in only 3% of the cases and not in a statistically significant manner. Inversely, the 19% reduction of BC recurrence appears to be associated with green tea drinking. However, green tea consumption was assessed in all the studies through questionnaire or interview, though only five studies used validated tools. Even though the food frequency questionnaires are cheaper and more manageable instruments to assess dietary intake, they cannot be considered devoid of potential bias. It was not possible to standardize the appropriate consumption value because green tea consumption was recorded in different units in the original studies. Indeed, tea consumption was reported in grams or cups. Furthermore, when cup unit was used as a reference, the amount of tea per cup differed, ranging from 100 mL to 350 mL. Due to this limitation, it was not possible to perform a dose–response analysis. In the absence of clear evidence answering questions about the right amount and frequency of consumption, it is not possible to estimate the amount of tea that may provide preventive effects. Indeed, a comparison between the highest intake of green tea, as reported in the original papers, with no consumption or lowest intake was performed. Nevertheless, a subgroup analysis that only included studies with green tea intake higher than 5 cups/day was carried out. Moreover, in order to minimize potential bias, adjusted data were collected from the original studies. In particular, attention was focused on adjustment for age, Body Mass Index (BMI), parity, history of BC, and smoking habit. Nonetheless, several studies also adjusted for other potential confounding factors such as age of menarche, age of first pregnancy, number of births, and menopausal status. 

This meta-analysis shows similar results previously obtained in another meta-analysis performed in 2010 [75]. However, in the study conducted by Ogunleye et al., only one database was consulted and a limited number of articles were retrieved. In addition, several subgroup analyses were performed.

### Strengths and Limitations of the Study

Some limitations have to be taken into account when reading this meta-analysis. First, the majority of the studies were conducted in Asia, mostly China and Japan, which represent the largest green tea production and consumption areas. Only one study was conducted in the USA, and no studies were conducted in Europe. This highlights the importance of performing similar studies in other ethnic populations in order to underline any potential differences. This is particularly true in determining whether other antioxidants consumed in the diet can play a confounding role. Another limitation is that self-reported green tea intake by FFQ was measured at baseline only, and the duration of tea consumption is not known. This can result in errors during both reporting and classification. Moreover, the different amounts of green tea consumed as higher intake in the original studies could have been defined in post hoc analysis. 

This is likely the first meta-analysis also assessing green tea intake, the risk of BC in post-menopausal women, and BC stage. Another significant strength of the present study is that the risk for BC and recurrence were considered both together and separately. However, due to the absence of information in the original studies, it was not possible to evaluate the effect of green tea based on BC stage at diagnosis. 

All the above-mentioned limitations can explain the different results between studies and the absence of statistical significance when looking at the different states of menopause or the preferable amount of intake. Future studies need to focus on these particular aspects. Moreover, studies with higher numbers of participants need to be performed. 

This meta-analysis has some important strengths, such as the large number of participants (163,810), with participants ranging from 20–87 years of age. Another strength is represented by the sensitivity analysis that was conducted in population subgroups. Furthermore, results in population subgroups are important for public health decision-makers to help them implement effective evidence-based strategies for selected population groups (e.g., high-risk participants), thereby enhancing the quality and efficiency of interventions [76]. 

According to the present results, the highest protective effect of green tea was found in BC recurrence prevention. This is in line with previous studies, where the authors demonstrated an anti-neoplastic effect of green tea through a reduction in viability and proliferation of BC cells [77,78,79]. In particular, a previous review on epigenetic modification by epigallocatechin on BC cell lines revealed a significantly greater inhibition of BC cell growth. Moreover, the inhibitory effect of epigallocatechin on DNA methyltransferase in BC cells is supposed to suppress proliferation through apoptosis induction as well [20]. Indeed, a meta-analysis evaluating the role of epigallocatechin in apoptosis induction in BC cell lines found a positive significant association with a ratio of mean (RoM) of 2.84 (95% CI 2.60–3.10) for BC cells treated with 20 μM of epigallocatechin [21].

Further epidemiological studies are needed to confirm these results and to quantify the impact of behavior change in this disease. Moreover, the majority of the included studies performed a satisfactory follow-up (a mean of approximately 10 years). 

## 5. Conclusion

A statistically significant inverse association between green tea and BC was found. This finding is in line with previous publications. However, the results are not always concordant, especially when looking at cohort studies or selecting only those studies analyzing the diagnosis of BC recurrence. However, a beneficial effect of green tea cannot be excluded, in particular because when only case-control studies were analyzed, a statistically significant protective effect was observed. Indeed, the importance of case-control studies in defining the causal relationship between exposure and event is well known. Therefore, further studies are required to confirm these data before any public recommendations can be made. In particular, it is important to conduct studies with greater numbers of people, in different countries, and with a serial evaluation of lifetime green tea consumption.

Moreover, as tea is one of the most popular beverages in the world and may possess many chemo-preventive qualities, a better understanding of the mechanisms might improve the utilization of green tea in breast cancer primary and recurrence prevention. At the clinical level, results from this systematic review and meta-analysis may be useful to design clinical trials aimed at ascertaining the potential role of green tea as an adjuvant in breast cancer therapy and pave the way to novel preventive strategies for breast cancer.

## Figures and Tables

**Table 1:** Search strategy details.

Search Strategy	Details
Inclusion criteria	P: general adult population (male and female) I: questionnaire or interview assessing green tea consumption C: higher vs. lower green tea consumption O: risk of breast cancer (if any) S: original research article (case-control studies, cohort studies, cross-sectional studies)
Exclusion criteria	P: pediatric population I: no administration of questionnaire O: other outcomes S: review article, expert opinion, comments, abstract, letters, article with no quantitative information or details
Language filter	English
Time filter	None (from inception)
Database	PubMed/Medline; Scopus; Web of Science

Abbreviations: P = Population; I = Intervention; C = Comparison; O = Outcome; S = Studies.

**Table 2:** Characteristics extracted from the included studies and quality score.

Author, Year	No in Analysis	Age (years)	Baseline	Study Period	Study Design	Instrument	Outcome	Green Tea Intake	OR RR HR (CI 95%)	*p*-Value	Country	QS/6
**Li M. et al. 2016**	Case = 756 Control = 789	20–84 y	BC diagnosis All stage	2011–2014	Case-control	In-person interview	recurrence	3 cups/day	1.2 (0.8–1.78)	0.38	China	4
**Iwasaki M. et al. 2014**	Case = 369 Control = 369	20–74 y	BC diagnosis 1–2 stage	2001–2005	Case-control	FFQ validated	recurrence	600 mL/day	1.27 (0.75–2.14)	0.20	Japan	5
**Iwasaki M. et al. 2010**	67,422	40–69 y	Healthy women	1990–1994 Follow-up 1995–1998	Prospective cohort	FFQ (self-administered) not validated	BC	5 cups/day	1.12 (0.81–1.56)	0.60	Japan	5
**Shrubsole J. M. et al. 2009**	Case = 3454 Control = 3474	25–70 y	BC diagnosis	1996–2005	Case-control	FFQ validated	recurrence	148 ± 124 g/mo	0.88 (0.79–0.98)	n.a.	China	4
**Inoue M. et al. 2008**	678	45–74 y	Healthy women	1993–1998	Prospective cohort	24 h food recalls validated	BC	174.6 ± 75.2 µg/day	1.00 (0.82–1.22)	0.41	China	4
**Zhang M. et al. 2007**	Case = 1009 Control = 1009	20–87 y	BC diagnosis	2004–2005	Case-control	FFQ validated	recurrence	4 cups/day	0.57 (0.47–0.69)	0.001	China	6
**Yuan J.M. et al. 2005**	665	45–74 y	Healthy women	1993–1998	Prospective cohort	In-person interview	BC	Weekly	0.91 (0.66–1.26)	n.a.	China	4
**Suzuki Y. et al. 2004 (a)**	17,353	>40 y (cohort I)	Healthy women	1984–1990	Prospective cohort	FFQ validated (self-administered)	BC	5 cups/day	0.96 (0.50–1.86)	0.51	Japan	6
**Suzuki Y. et al. 2004 (b)**	24,769	40–64 y (cohort II)	Healthy women	1984–1990	Prospective cohort	FFQ validated (self-administered)	BC	5 cups/day	0.85 (0.43–1.669)	0.95	Japan	6
**Wu H. A. et al. 2003**	Case = 501 Control = 594	25–74 y	BC diagnosis	1995–1998	Case-control	In-person interview	recurrence	85.7 mL/day	0.61 (0.40–0.93)	0.01	USA	4
**Inoue M. et al. 2001**	1160	Mean age 51.5 y	BC diagnosis 1–2 stage	1990–1997	Follow-up	FFQ (self-administered) not validated	recurrence	6 cups/day	0.68 (0.4–1.16)	0.72	Japan	3
**Nagano J. et al. 2001**	38,540	Mean age 54.8 y	Healthy women	1979–1981	Prospective cohort	FFQ (self-administered) not validated	BC and other cancers	5 cups/day	1.0 (0.67–1.6)	0.80	Japan	3
**Key T.J. et al. 1999**	34,759	40–80 y	Healthy women	1969–1981	Prospective cohort	FFQ not validated	BC	5 cups/day	0.86 (0.62–1.21)	0.284	Japan	3
**Nakachi K. et al. 1998**	472	Mean age 49.7±11.2	BC diagnosis 1–2–3 stage	1984–1993	Follow-up	FFQ not validated	recurrence	8 cups/day	0.775 (0.53–1.13)	0.15	Japan	4

Abbreviations: QS = quality score; BC = breast cancer; FFQ = food frequency questionnaire; n.a.= not available; OR = odds ratio; RR = Relative Risk; HR = Hazard Ratio

**Table 3:** Data extracted from primary studies focusing on menopausal status.

	Premenopausal		Postmenopausal	
Author, Year	N in Analysis	OR RR HR (CI 95%)	N in Analysis	OR RR HR (CI 95%)
Li M. et al. 2016	267	OR 0.62 (0.40–0.97)	405	OR 1.40 (1.00–1.96)
Iwasaki M. et al. 2014	79	OR 1.10 (0.54–2.23)	212	OR 1.42 (0.71–2.85)
Iwasaki M. et al. 2010	81	HR 0.97 (0.66–1.41)	70	HR 1.08 (0.75–1.55)
Shrubsole M. et al. 2009	1302	0R 0.87 (0.76–1.00)	799	OR 0.88 (0.74–1.04)

Abbreviations: N = number; OR = odds ratio; RR = Relative Risk; HR = Hazard Ratio; CI = confidence interval

## Supplementary Materials

The following are available online at http://www.mdpi.com/2072-6643/10/12/1886/s1. Table S1: Studies excluded with reason.
