# Supplementary material for: Green Tea Consumption and Risk of Breast Cancer and Recurrence—A Systematic Review and Meta-Analysis of Observational Studies
Source: Nutrients. 2018 Dec 3;10(12):1886. doi: 10.3390/nu10121886 (PMC6316745; doi:10.3390/nu10121886)
Supplement: Supplementary file 1 [file nutrients-10-01886-s001.pdf]

Table S1: Studies excluded with reason.

Author and year	Reason of exclusion
Arthur et al., 2018<br>Ronco et al., 2016<br>Bhoo-Pathy et al., 2015<br>Oh et al., 2015<br>Touvier et al., 2012<br>Fagherazzi et al., 2011<br>Boggs et al., 2010<br>Luo et. al., 2010<br>Luo et al., 2012<br>Pathy et al., 2010<br>Michels et al., 2002<br>Kumar et al., 2009<br>Ganmaa et al., 2008<br>Zheng et al., 1996<br>McLaughlin et al., 1991<br>Wang et al., 2014	No differentiation between green tea and others
Crew et al., 2012<br>Crew et al., 2015<br>Lazzeroni et al., 2017<br>Samavat et al., 2015<br>Samavat et al., 2017	No food dietary intake but extract supplementation
Chen et al., 2010<br>Stendell-Hollis et al., 2010<br>Zhu et al., 2016	Different outcome
Mayo Clinic, 2008	Full text not available
Iwasaki et al., 2010<br>Zhang et al., 2009	Data not available
